# Longitudinal association between social interaction and screen-positive cognitive impairment risk in older adults: domain-specific effects and the impact of baseline cognitive status

**DOI:** 10.3389/fpubh.2026.1873956

**Published:** 2026-10-06

**Authors:** Shuanghong Li, Akihiro Kakuda, Yuko Sawada, Jinrui Zhang, Maiko Shigeeda, Zhizheng Zhang, Meiling Qian, Ruifeng Zhao, Haotian Gao, Hiroshi Kinoshita, Rika Okumura, Yuriko Kawasaki, Tokie Anme

**Affiliations:** 1 Graduate School of Comprehensive Human Science, University of Tsukuba, Tsukuba, Japan; 2 Department of Physical Therapy, Morinomiya University of Medical Sciences, Osaka, Japan; 3 Department of Public Welfare, Tobishima, Japan; 4 Faculty of Medicine, University of Tsukuba, Tsukuba, Ibaraki, Japan

**Keywords:** longitudinal study, multiple imputation, older adults, screen-positive cognitive impairment risk, social interaction

## Abstract

**Background:**

Social relationships are key social determinants of healthy aging. However, the population-level impact of different dimensions of social interaction and their dependence on baseline cognitive status remain insufficiently understood.

**Methods:**

We used a two-wave longitudinal dataset (2020–2023) of community-dwelling older adults (*N* = 418). Social interaction was assessed using the Index of Social Interaction, covering five domains: independence, social curiosity, interaction, participation in society, and feeling of safety. The outcome was a self-reported Kihon Checklist screen-positive cognitive impairment risk classification rather than a clinical diagnosis. Baseline-cognition-adjusted prospective regression models were designated as the principal analyses, and models without baseline cognition were secondary. Missing values in the derived analytic variables were handled using multiple imputation with 40 completed datasets and pooling according to Rubin’s rules. Linear-model inference used HC3 heteroscedasticity-robust standard errors, and inverse-probability-of-attrition weighting (IPAW) was used as an attrition sensitivity analysis.

**Results:**

In the principal baseline-cognition-adjusted model, baseline total ISI was not independently associated with 2023 screen-positive cognitive impairment risk (OR = 0.92, 95% CI 0.79–1.08, *p* = 0.309), and none of the five ISI domains was significant. Baseline screen-positive cognitive impairment risk was strongly associated with follow-up screening status (total-ISI model OR = 8.05, 95% CI 4.91–13.21, *p* < 0.001). Secondary models without baseline cognition showed inverse associations for total ISI (OR = 0.83, 95% CI 0.72–0.96, *p* = 0.011) and social curiosity (OR = 0.72, 95% CI 0.56–0.92, *p* = 0.008), but these did not persist in the principal adjusted analyses. Stabilized IPAW yielded a non-significant total-ISI estimate (OR = 0.896, 95% CI 0.797–1.008, *p* = 0.067); 1st–99th percentile truncation yielded OR = 0.901 (95% CI 0.800–1.015, *p* = 0.086).

**Conclusion:**

After accounting for baseline cognitive screening status, we found no statistically significant independent longitudinal association between baseline social interaction and subsequent screen-positive cognitive impairment risk. The attenuation of secondary unadjusted associations suggests that pre-existing cognitive differences and reverse directionality are important considerations. Social-interaction domains should therefore be interpreted as exploratory correlates rather than established protective or preventive targets.

## 1 Introduction

Globally, the number of people with dementia has surpassed 50 million, a figure projected to increase to 82 million by 2030 and reach 152 million by 2050. This will impose profound economic burdens and productivity losses on governments, communities, families, and individuals. In both low- and high-income countries, dementia is a leading cause of dependency and disability among older adults, ranking among the top 10 causes of healthy life years lost to disability in individuals aged 60 years and older (1, 2).

This challenge is particularly severe in Japan, which has one of the most rapidly aging populations in the world. Previous estimates indicate that the societal cost of dementia in Japan was approximately 14.5 trillion JPY in 2014, with projections suggesting a potential increase to 24.3 trillion JPY by 2060, driven primarily by long-term and informal care expenditures (3). Population-level dementia prevalence may also vary across successive cohorts and calendar periods, emphasizing the importance of temporal context when interpreting population estimates (4).

In the absence of curative therapies, modifiable factors associated with cognitive health must be identified. Social relationships have attracted increasing attention as potential protective factors against cognitive decline and dementia (5). Meta-analytic evidence indicates that poor social relationships are associated with a higher risk of dementia and cognitive decline (6, 7). Specifically, lower social participation, less frequent social contact, and higher levels of loneliness have all been linked to an increased risk of dementia, whereas broader aspects of poor social relationships are associated with subsequent cognitive decline (8–10).

Previous research has often combined distinct aspects of social connectedness. The ISI primarily captures self-reported behavioral and functional engagement (e.g., information seeking, interaction, social participation, and availability of support), whereas loneliness and perceived social isolation represent subjective emotional experiences (11, 12). A recent multidimensional preprint distinguishes relatively objective or structural features of connectedness, such as social-network size and cohabitation, from subjective loneliness (13). These dimensions are related but not interchangeable and separating them may help clarify which social processes are associated with cognitive health. Longitudinal associations may also depend strongly on whether baseline cognitive status is controlled. Because the present study has only two waves, it is positioned as a preliminary prospective analysis rather than a design capable of separating stable between-person differences from within-person change (14, 15).

In this study, we examined prospective associations between social interaction in 2020 and screen-positive cognitive impairment risk in 2023 using the total ISI score and five domains. Baseline-cognition-adjusted models were designated as the principal analyses in the revised framework, with unadjusted models presented secondarily. We additionally evaluated selective attrition and robust inference. The aim is to characterize longitudinal associations cautiously rather than to infer that social interaction prevents cognitive decline.

## 2 Methods

### 2.1 Study design and participants

This study used a two-wave prospective design based on data collected from community-dwelling older adults in Japan between 2020 and 2023. A total of 845 adults aged 60 years or older were initially identified. At baseline in 2020, the ISI and cognitive-risk information were collected during the same survey period using two separate questionnaire forms. Construction of the linked baseline dataset required a participant record from both forms. Accordingly, 157 individuals who did not complete one of the two baseline forms could not be matched across the two questionnaires and were not included in the linked baseline analytic cohort, leaving 688 baseline participants. Of these, 270 (39.2%) had no 2023 follow-up and were considered lost to follow-up, leaving 418 participants with follow-up participation (Figure 1). Participants within the retained cohort could still have missingness in the derived analytic variables and remain eligible for multiple imputations. The study protocol was approved by the Medical Ethics Committee, Faculty of Medicine, University of Tsukuba, Japan (Approval No. 1331-7), and the study was conducted in accordance with local legislation and institutional requirements.

**Figure 1:**
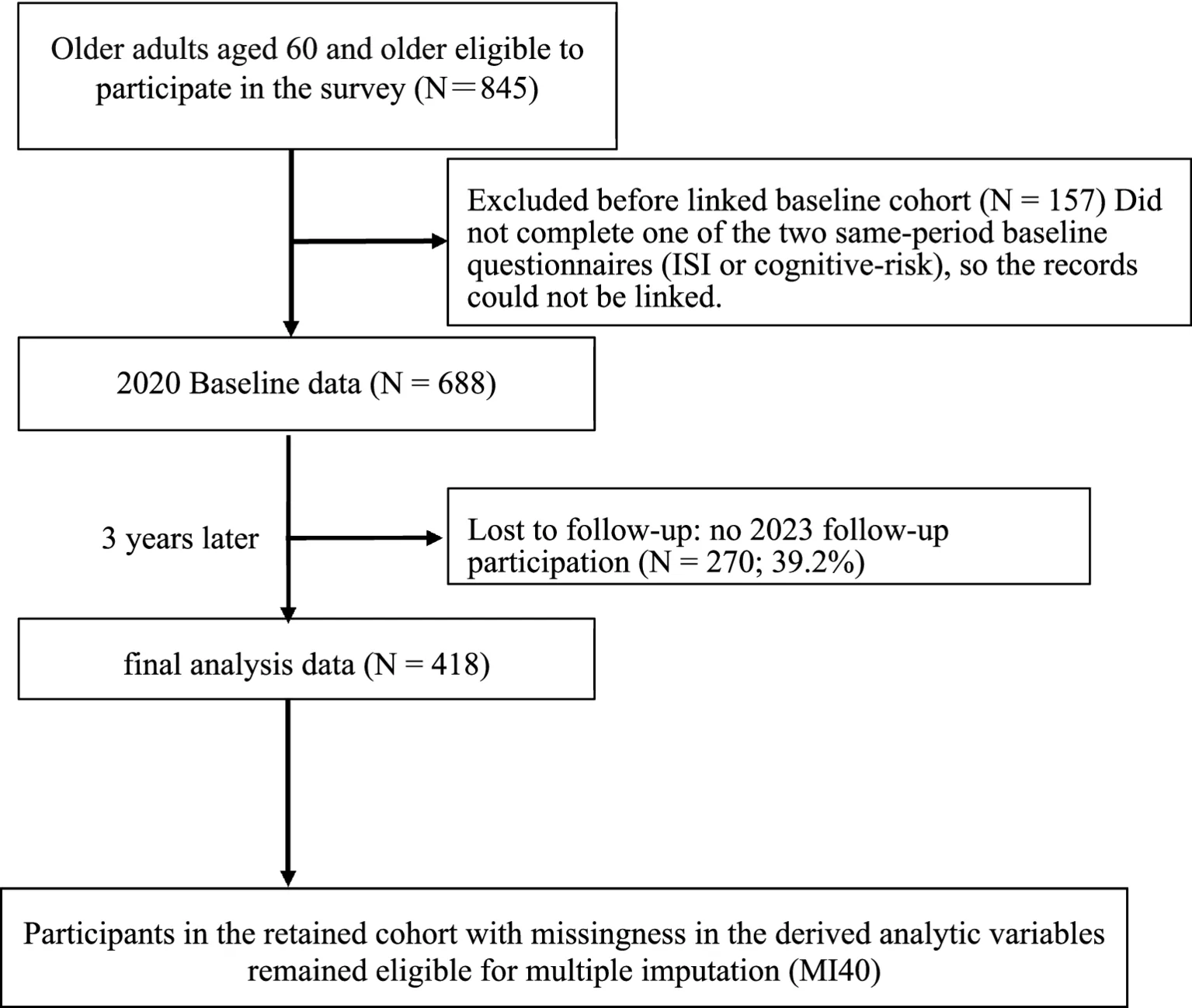
Participant flow chart in 2020–2023.

### 2.2 Measures

Social interaction was evaluated using the Index of Social Interaction (ISI), an 18-item scale applicable to individuals aged 60 and above. The five domains are independence (4 items), social curiosity (5 items), interaction (3 items), participation in the society (4 items), and feeling of safety (2 items). Each item is scored dichotomously, with higher domain and total scores indicating greater social interaction/engagement. The domains are treated as behavioral/functional indicators and should not be equated with subjective loneliness or perceived isolation (16).

Screen-positive cognitive impairment risk was assessed using the cognitive category of the Kihon Checklist, a self-report screening instrument used in Japan. The cognitive category comprises items 18–20; participants with at least one adverse response were classified as screen-positive. This classification is a screening indicator and not a clinical diagnosis, dementia endpoint, or direct measure of objective cognitive decline. Japanese validation studies support the prognostic relevance of this three-item category. In a cohort of 13,974 community-dwelling Japanese adults aged ≥65 years, a KCL cognitive-function cutoff of ≥1 had sensitivity of 60.2%, specificity of 65.1%, and an AUC of 0.65 (95% CI 0.63–0.66) for incident dementia, indicating useful but limited discrimination (17). Another Japanese validation study found associations between the KCL cognitive category and objective Sweet 16 performance (18). We therefore use screening terminology throughout and do not equate the measure with clinically diagnosed cognitive impairment.

Baseline covariates, all measured in 2020, included sex, age, living status, subjective economic status, care status, chronic disease status, physical activity, alcohol consumption, and smoking status. These variables were selected because of established associations with cognitive health (19, 20). Age was entered continuously. Binary variables used the stored 0/1 analysis coding with 0 as the regression reference. Sex was coded 0 = female and 1 = male. Living status was coded 0 for living alone and 1 for any cohabiting household arrangement. Subjective economic status was coded 0 for responses of ‘very difficult’ or ‘somewhat difficult’ and 1 for ‘ordinary,’ ‘somewhat comfortable,’ or ‘very comfortable.’ Care status was coded 0 when no care or assistance was needed and 1 when some care or assistance was needed, regardless of whether it was currently being received. Chronic disease status was coded 1 when at least one of the questionnaire-listed conditions currently under treatment or with sequelae was reported and 0 when ‘none’ was selected. Physical activity was coded 1 for exercise at least 1–2 times per week (including 1–2 times/week, 3–4 times/week, or almost every day) and 0 for ‘hardly ever.’ Alcohol consumption was coded 1 for responses of ‘almost every day’ or ‘sometimes’ and 0 for ‘hardly ever’ or ‘never.’ Smoking status was coded 1 for current smoking (‘almost every day’ or ‘sometimes’) and 0 for former smoking or never smoking. The complete original response categories and derivation rules are provided in Supplementary Table S7.

### 2.3 Handling of missing data

Missing values in the derived analytic variables among retained participants were addressed using multiple imputations by chained equations (MICE), with 40 completed datasets and pooling according to Rubin’s rules (21, 22). Missingness reached 26.6% for follow-up total ISI. Little’s MCAR test was significant (*χ*^2^ = 550.504, df = 420, *p* < 0.001), indicating that the observed missing-data pattern was not consistent with MCAR (23). Accordingly, missing at random (MAR) was treated as an analytic assumption rather than an empirically proven mechanism. Because missingness exceeded 25% for a key social-interaction measure, the number of imputations was increased from 20 to 40 to reduce Monte Carlo variability and improve the stability of pooled standard errors and confidence intervals.

The final MI40 procedure was implemented in Python using the MICEData class in the statsmodels package. Forty complete datasets were generated after 20 burn-in update cycles, with five additional chained-equation update cycles between consecutively saved imputations and a random seed of 20260818. Imputation was performed on the derived analytic variables rather than on the 18 individual ISI item responses. The imputation model included baseline and follow-up total ISI scores, all five derived ISI domain scores, baseline and follow-up cognitive-risk indicators, and all baseline covariates included in the analytic models.

The default MICEData conditional-imputation framework with predictive mean matching was used rather than variable-specific logistic models for binary variables or ordinal models for discrete bounded scores. These variable-specific approaches were considered but were not implemented in the final MI40 datasets. The default framework was retained to provide a consistent imputation procedure across analytic variables and to maintain consistency with the MI40 datasets used for all reported analyses.

After each saved imputation, deterministic post-processing was applied to restore the intended coding of the analytic variables. Binary variables, including cognitive-risk indicators and binary covariates, were converted to 0 or 1 using a threshold of 0.5. ISI domain scores were rounded to integer values, with a generic 0–5 bound applied in the implemented post-processing procedure, and total ISI scores were constrained to the valid range of 0–18. Although the same generic 0–5 bound was applied to the domain scores, inspection of the completed datasets confirmed that all imputed domain scores remained within their respective theoretically valid ranges: independence, 0–4; social curiosity, 0–5; interaction, 0–3; participation in society, 0–4; and feeling of safety, 0–2. These post-processing steps served as coding safeguards against non-integer or out-of-range values following imputation. No interaction or nonlinear terms were specified in the imputation models.

Because total ISI and the five derived domain scores were imputed as separate variables, exact algebraic equality between an imputed total ISI score and the sum of the five imputed domain scores was not passively enforced. Total-score and domain-specific regression models were therefore fitted separately, and mathematically related ISI measures were not simultaneously entered as exposures in the same outcome model. The absence of passive score-constraint imputation is acknowledged as a limitation of the imputation procedure.

Stability of the imputed distributions was examined across the 40 completed datasets using trace summaries of variable means and standard deviations. The same 40 complete datasets were used for principal and secondary outcome models. MICE addressed missingness in the derived analytic variables among retained participants and did not recover the 270 participants with no 2023 follow-up. For IPAW, baseline predictors in the full 688-person cohort were also multiply imputed 40 times; retention status was fully observed, was not imputed, and served as the outcome of the retention model.

### 2.4 Statistical analysis

The revised analysis framework used prospective regression rather than cross-lagged panel terminology. Principal cognitive-outcome models were logistic regressions predicting 2023 screen-positive cognitive impairment risk from baseline total ISI (or one ISI domain), baseline cognitive screening status, and baseline covariates. Secondary models omitted baseline cognitive screening status. Linear regressions predicted follow-up ISI/domain scores from the corresponding baseline score and covariates; reciprocal-direction models additionally included baseline cognitive screening status. For linear outcomes, HC3 heteroscedasticity-consistent covariance matrices were estimated within each of the 40 imputed datasets and then pooled using Rubin’s rules. Total ISI was designated as the primary exposure. The five domain-specific analyses were exploratory; no confirmatory claim was based on domain-wise multiplicity, and domain results were interpreted cautiously rather than as independent effects. Because domain score ranges differ, standardized per-SD ORs were estimated within each imputed dataset and pooled for descriptive comparison (Supplementary Table S6). Complete case analyses were conducted as missing-data sensitivity analyses.

Linear-model residual normality and heteroscedasticity were examined using Jarque–Bera (24) and Breusch–Pagan (25) diagnostics. Because heteroscedasticity was detected for several bounded ISI outcomes, HC3 heteroscedasticity-consistent inference was used for the final MI40 linear models (26). Logistic-model discrimination and calibration were treated as distinct properties. AUC was used for discrimination, whereas Hosmer–Lemeshow statistics were treated only as imputation-specific goodness-of-fit/calibration diagnostics and were not averaged across imputed datasets (27). Across the MI40 baseline-cognition-adjusted logistic models, mean AUC values ranged from 0.784 to 0.791 and mean Brier scores were approximately 0.176; corresponding secondary models without baseline cognition showed limited discrimination (mean AUC 0.625–0.646; mean Brier score 0.223–0.226). A representative complete-case principal total-ISI model had an apparent AUC of 0.815 (bootstrap 95% CI approximately 0.758–0.867) and Brier score 0.165. These are descriptive apparent-performance measures rather than pooled out-of-sample validation. For attrition sensitivity analysis, retention probability was modeled among all 688 baseline participants from age, sex, living status, subjective economic status, care status, chronic disease, physical activity, alcohol use, smoking, baseline cognitive screening status, and total ISI. Stabilized IPAW was calculated as the marginal probability of retention divided by the individual predicted retention probability; 1st–99th percentile truncated weights were also examined (28). Weight diagnostics included effective sample size and standardized mean differences before and after weighting.

SPSS Statistics 30 (IBM Corporation, Armonk, NY, USA) was used for data checking, descriptive statistics, Little’s MCAR test, and missing-data pattern inspection. Python 3.11 (Python Software Foundation, Wilmington, DE, USA), with pandas, NumPy, SciPy, statsmodels (29), and scikit-learn (30), was used for MI40 generation, prospective regression, HC3 robust inference, standardized per-SD analyses, IPAW, model diagnostics, and data management. Visual Studio Code (Microsoft Corporation, Redmond, WA, USA) was used solely as the development environment.

## 3 Results

### 3.1 Participant characteristics and missingness

Among the 418 participants with follow-up participation, mean total ISI was 16.48 (SD = 2.05) in 2020 and 16.46 (SD = 2.06) in 2023. The prevalence of screen-positive cognitive impairment risk was approximately 60% at baseline and 61% at follow-up. At follow-up, 241 participants were observed as screen-positive, 151 as screen-negative, and 26 had missing cognitive outcome data. Across the 40 complete datasets used for the principal logistic models, the mean event count was 254.9, and the mean non-event count was 163.1. The total-ISI model contained 11 predictor terms, corresponding descriptively to approximately 23.2 events and 14.8 non-events per predictor term; this is reported as sample-adequacy context rather than as a formal power guarantee. Missingness in the derived analytic variables varied substantially across social interaction measures (Table 1).

**Table 1:** Descriptive statistics of the study variables (*N* = 418).

Variable	*n*	Missing (%)	*M*	SD
Social interaction				
ISI (2020, total score)	314	24.88	16.48	2.05
Independence (2020)	400	4.31	3.92	0.43
Social curiosity (2020)	390	6.70	4.28	1.06
Interaction (2020)	398	4.78	2.84	0.45
Participation in the society (2020)	333	20.33	3.46	0.72
Feeling of safety (2020)	398	4.78	1.86	0.45
ISI (2023, total score)	307	26.56	16.46	2.06
Independence (2023)	400	4.31	3.92	0.39
Social curiosity (2023)	375	10.29	4.25	1.08
Interaction (2023)	388	7.18	2.84	0.46
Participation in the society (2023)	363	13.16	3.44	0.78
Feeling of safety (2023)	409	2.15	1.89	0.40
Cognitive function				
Screen-positive cognitive impairment risk (2020)	403	3.59	0.60	0.49
Screen-positive cognitive impairment risk (2023)	392	6.22	0.61	0.49
Covariates				
Sex	418	0.00	0.46	0.50
Age	418	0.00	73.13	6.47
Living status	414	0.96	0.91	0.29
Subjective economic status	399	4.55	0.81	0.39
Care status	414	0.96	0.08	0.27
Chronic disease status	407	2.63	0.82	0.39
Physical activity	385	7.89	0.64	0.48
Alcohol consumption	417	0.24	0.37	0.48
Smoking status	416	0.48	0.08	0.28

Values are presented as the means (*M*) and standard deviations (SD). Missing (%) represents the percentage of missing observations. Screen-positive cognitive impairment risk (CIR) was operationalized as a binary variable (0 = no risk, 1 = at risk). ISI refers to the Index of Social Interaction, comprising five domains: independence, social curiosity, interaction, participation in the society, and feeling of safety.

### 3.2 Principal finding: no independent association after baseline cognitive adjustment

In the principal baseline-cognition-adjusted logistic model pooled across 40 imputations, higher baseline total ISI was not significantly associated with 2023 screen-positive cognitive impairment risk (OR = 0.92, 95% CI 0.79–1.08, *p* = 0.309). Baseline cognitive screening status was strongly associated with follow-up screening status (total-ISI model OR = 8.05, 95% CI 4.91–13.21, *p* < 0.001). None of the five ISI domains was statistically significant after baseline cognitive status and covariates were included: independence OR = 0.60 (95% CI 0.21–1.66, *p* = 0.323), social curiosity OR = 0.81 (95% CI 0.61–1.08, *p* = 0.155), interaction OR = 1.13 (95% CI 0.63–2.04, *p* = 0.675), participation in the society OR = 1.03 (95% CI 0.73–1.44, *p* = 0.871), and feeling of safety OR = 0.80 (95% CI 0.46–1.42, *p* = 0.449) (Table 2; Supplementary Tables S5A-S5F).

**Table 2:** Principal MI40 logistic regression models adjusted for baseline cognitive screening status.

Exposure	OR	95% CI	*p*	Baseline cognition OR	95% CI	*p*
Total ISI	0.92	0.79–1.08	0.309	8.05	4.91–13.21	<0.001
Independence	0.60	0.21–1.66	0.323	8.17	5.00–13.36	<0.001
Social curiosity	0.81	0.61–1.08	0.155	8.03	4.90–13.14	<0.001
Interaction	1.13	0.63–2.04	0.675	8.62	5.21–14.26	<0.001
Participation in the society	1.03	0.73–1.44	0.871	8.42	5.16–13.74	<0.001
Feeling of safety	0.80	0.46–1.42	0.449	8.32	5.10–13.58	<0.001

MI40 pooled logistic regression adjusted for baseline cognitive screening status and baseline covariates (age, sex, living status, subjective economic status, care status, chronic disease status, physical activity, alcohol consumption, and smoking status). ORs are per one-unit increase in each exposure. Cognitive screening status was coded 0 = screen-negative and 1 = screen-positive. *p* values are shown to 3 decimals; all other statistical estimates to 2 decimals. CI, confidence interval; ISI, Index of Social Interaction; OR, odds ratio.

The MI40 pooled HC3 analyses confirmed statistically significant baseline-to-follow-up stability for total ISI (*B* = 0.62, 95% CI 0.50–0.74, *p* < 0.001), independence (*B* = 0.48, 95% CI 0.20–0.75, *p* < 0.001), social curiosity (*B* = 0.57, 95% CI 0.45–0.69, *p* < 0.001), interaction (*B* = 0.27, 95% CI 0.11–0.43, *p* = 0.001), participation in the society (*B* = 0.39, 95% CI 0.28–0.51, *p* < 0.001), and feeling of safety (*B* = 0.39, 95% CI 0.19–0.60, *p* < 0.001) (Table 3). In reciprocal-direction models, baseline cognitive screening status was not significantly associated with follow-up total ISI, independence, interaction, participation in the society, or feeling of safety; a small inverse association was observed for social curiosity (*B* = −0.18, 95% CI − 0.33 to −0.03, *p* = 0.018) (Supplementary Table S3).

**Table 3:** MI40 HC3 linear models for follow-up ISI scores and reciprocal-direction associations.

Outcome	Baseline same-score *B*	95% CI	*p*	Baseline cognitive status *B*	95% CI	*p*
Total ISI	0.62	0.50–0.74	<0.001	−0.12	−0.39 to 0.16	0.405
Independence	0.48	0.20–0.75	<0.001	−0.04	−0.09 to 0.01	0.157
Social curiosity	0.57	0.45–0.69	<0.001	−0.18	−0.33 to −0.03	0.018
Interaction	0.27	0.11–0.43	0.001	−0.03	−0.12 to 0.06	0.495
Participation in the society	0.39	0.28–0.51	<0.001	0.00	−0.14 to 0.14	0.991
Feeling of safety	0.39	0.19–0.60	<0.001	0.00	−0.07 to 0.07	0.958

MI40 pooled linear regression with HC3 heteroscedasticity-robust inference. Each model included the corresponding baseline ISI score, baseline cognitive screening status, and baseline covariates (age, sex, living status, subjective economic status, care status, chronic disease status, physical activity, alcohol consumption, and smoking status). *B* denotes an unstandardized regression coefficient. *p* values are shown to 3 decimals; all other statistical estimates to 2 decimals. ISI, Index of Social Interaction.

Because the principal domain-specific models are separate exploratory regressions, these results do not establish an independent effect of any single domain after accounting for the others. Standardized per-SD ORs from the principal baseline-adjusted models ranged from 0.81 to 1.06, and all 95% CIs included 1; they are therefore reported as descriptive effect-size aids in Supplementary Table S6.

### 3.3 Secondary findings without baseline cognitive adjustment

In secondary MI40 models that did not include baseline cognitive screening status, higher total ISI was associated with lower follow-up screening risk (OR = 0.83, 95% CI 0.72–0.96, *p* = 0.011), and social curiosity was the only domain with a statistically significant inverse association (OR = 0.72, 95% CI 0.56–0.92, *p* = 0.008). Independence (OR = 0.42, *p* = 0.107), interaction (OR = 0.66, *p* = 0.144), participation in the society (OR = 1.01, *p* = 0.940), and feeling of safety (OR = 0.69, *p* = 0.152) were not significant (Table 4). These secondary associations were attenuated in the principal baseline-adjusted models and are not interpreted as causal or protective effects.

**Table 4:** Secondary MI40 logistic regression models without baseline cognitive screening status.

Exposure	OR	95% CI	*p*
Total ISI	0.83	0.72–0.96	0.011
Independence	0.42	0.15–1.20	0.107
Social curiosity	0.72	0.56–0.92	0.008
Interaction	0.66	0.38–1.15	0.144
Participation in the society	1.01	0.75–1.37	0.940
Feeling of safety	0.69	0.41–1.15	0.152

MI40 pooled logistic regression without baseline cognitive screening status, adjusted for baseline covariates (age, sex, living status, subjective economic status, care status, chronic disease status, physical activity, alcohol consumption, and smoking status). ORs are per one-unit increase in each exposure. *p* values are shown to 3 decimals; all other statistical estimates to 2 decimals. CI, confidence interval; ISI, Index of Social Interaction; OR, odds ratio.

Attrition and missing-data sensitivity analyses indicated that the 270 participants lost to follow-up differed from the 418 retained participants on several baseline characteristics. Those lost were older, had lower total ISI, lower social curiosity, lower interaction, lower participation-in-the-society scores, greater care needs, and less physical activity; baseline cognitive screening status did not differ significantly (Supplementary Table S1). In the MI40 IPAW analysis, stabilized weights were well behaved (mean = 1.003; maximum = 3.971), the mean effective sample size remained 387.6, and weighting reduced all modeled baseline absolute SMDs to below 0.04. The total-ISI estimate remained non-significant after stabilized IPAW (OR = 0.896, 95% CI 0.797–1.008, *p* = 0.067) and 1st–99th percentile truncation (OR = 0.901, 95% CI 0.800–1.015, *p* = 0.086); none of the five weighted domain estimates was significant (Supplementary Table S2). Complete-case baseline-cognition-adjusted analyses likewise showed no significant total-ISI association (OR = 0.860, 95% CI 0.692–1.069, *p* = 0.173), although social curiosity was significant in the complete-case model (OR = 0.685, 95% CI 0.478–0.981, *p* = 0.039), indicating some sensitivity of this exploratory domain estimate to missing-data handling (Table 5; Supplementary Table S4).

**Table 5:** Standardized and complete-case sensitivity analyses.

Exposure	Per-SD OR (MI40 principal)	95% CI	*p*	Complete-case OR	95% CI	*p*
Total ISI	0.86	0.63–1.16	0.309	0.86	0.69–1.07	0.173
Independence	0.81	0.53–1.24	0.323	0.64	0.24–1.73	0.380
Social curiosity	0.81	0.60–1.09	0.155	0.68	0.48–0.98	0.039
Interaction	1.06	0.81–1.38	0.675	0.87	0.44–1.74	0.701
Participation in the society	1.02	0.79–1.32	0.870	0.80	0.50–1.28	0.357
Feeling of safety	0.91	0.70–1.17	0.449	0.76	0.40–1.44	0.398

Per-SD estimates are from MI40 principal baseline-cognition-adjusted logistic models. Complete-case ORs are per one-unit increase in each exposure. *p* values are shown to 3 decimals; all other statistical estimates to 2 decimals. CI, confidence interval; ISI, Index of Social Interaction; OR, odds ratio.

### 3.4 Robustness and model performance

All MI40 logistic models produced estimable pooled results. In the baseline-cognition-adjusted models, mean AUC values across the six exposure specifications ranged from 0.784 to 0.791 and mean Brier scores were approximately 0.176, whereas secondary models without baseline cognition had limited discrimination (mean AUC 0.625–0.646) and higher Brier scores (approximately 0.223–0.226). A representative complete-case principal total-ISI model had an apparent AUC of 0.815 (bootstrap 95% CI approximately 0.758–0.867) and Brier score 0.165. AUC is interpreted as discrimination, whereas calibration concerns agreement between predicted and observed probabilities; imputation-specific Hosmer–Lemeshow results were not averaged to create a pooled calibration claim. The representative complete-case metrics are descriptive apparent performance and not pooled external or cross-validated performance.

Breusch–Pagan diagnostics indicated heteroscedasticity for several bounded ISI outcomes. Re-estimation using HC3 robust covariance within each of the 40 imputed datasets did not materially change the point estimates and retained statistically significant baseline-to-follow-up stability for total ISI and all five domains. Complete case HC3 analyses showed the same overall direction. HC3 addresses heteroscedasticity in statistical inference but does not eliminate limitations arising from bounded, discrete score distributions or ceiling effects (31).

## 4 Discussion

Using a two-wave prospective design, we examined whether baseline social interaction was associated with subsequent screen-positive cognitive impairment risk. In the final MI40 principal analyses, adjustment for baseline cognitive screening status yielded no evidence of an independent association for total ISI (OR = 0.922, 95% CI 0.787–1.079) or any of its five domains. Baseline cognitive screening status, in contrast, was strongly associated with follow-up screening status (total-ISI model OR = 8.051, 95% CI 4.908–13.209). Secondary models without baseline cognition showed inverse associations for total ISI and social curiosity, but these were attenuated after baseline cognition was included.

The attenuation after baseline cognitive adjustment suggests that previously observed inverse associations may reflect, at least partly, pre-existing cognitive differences or reverse directionality. Individuals with better baseline cognition may be more able or inclined to sustain information-seeking, hobbies, communication, and other social-cognitive activities. Accordingly, the present findings should not be interpreted as evidence that increasing social interaction independently prevents later cognitive impairment. This cautious interpretation is consistent with literature on active and socially integrated lifestyles in later life (32), while age-related cognitive changes can begin before overt clinical impairment (33).

The domain-specific results require particular caution. Social curiosity was inversely associated with the outcome in the secondary model without baseline cognition and in the complete-case sensitivity analysis, but not in the MI40 principal model or either IPAW model; thus, this domain-specific association was not robust to analytic specification. A reciprocal-direction MI40 HC3 model showed a modest inverse association between baseline cognitive screening risk and later social curiosity, which is compatible with the possibility that pre-existing cognitive differences influence subsequent cognitively stimulating engagement. Separate domain models do not demonstrate domain independence, and one-point ORs are not directly comparable across domains with different score ranges. We therefore characterize all domain-specific analyses as exploratory. The findings should also be interpreted within the broader context of multidimensional geriatric vulnerability. Social and material disadvantages may cluster with adverse nutritional and other health conditions in older adults (34), suggesting that cognitive vulnerability may coexist with other geriatric syndromes rather than occur in isolation. In addition, the ISI independence domain may capture not only aspects of social engagement but also broader health-related and functional characteristics. Evidence that independence in activities of daily living is associated with health and lifestyle factors (35) further supports caution in interpreting this domain as a purely social construct.

Selective attrition was evident: participants lost to follow-up were older and showed less favorable baseline social and health profiles on several measures. The MI40 stabilized-IPAW procedure produced well-behaved weights (mean 1.003; maximum 3.971), preserved a mean effective sample size of 387.6, and reduced all modeled baseline absolute SMDs to below 0.04. Stabilized and truncated IPAW produced similar total-ISI estimates (OR = 0.896 and 0.901, respectively), both with confidence intervals including 1. These findings suggest that adjustment for observed predictors of attrition did not materially alter the principal conclusion, although bias from unmeasured determinants of follow-up cannot be excluded.

Baseline screen-positive cognitive impairment risk was strongly associated with subsequent screening status (OR = 8.051 in the principal total-ISI model and OR = 8.892 in the stabilized-IPAW total-ISI model). We deliberately avoid describing this as the “strongest predictor,” because predictors were not compared on a common standardized scale. Instead, the finding highlights the importance of accounting for initial cognitive screening status when evaluating prospective social-interaction associations.

## 5 Limitations

Several limitations should be considered when interpreting our findings.

First, with only two measurement waves, the prospective regressions cannot separate stable between-person differences from within-person change, estimate a random-intercept cross-lagged structure, or establish causal direction. Reciprocal-direction regressions provide complementary information but are not equivalent to a conventional CLPM or RI-CLPM.

Second, the cognitive outcome is a self-reported three-item Kihon Checklist screening classification rather than a clinical diagnosis or comprehensive objective cognitive assessment. It cannot quantify the magnitude or trajectory of cognitive decline. Cognitive impairment without dementia is a recognized epidemiological construct, but the present KCL screening classification should not be equated with such a clinical/epidemiological diagnosis (36). Different psychometric tests also vary in their sensitivity to longitudinal cognitive change (37). In a large Japanese cohort, the ≥1 KCL cognitive-function cutoff showed sensitivity of 60.2%, specificity of 65.1%, and AUC of 0.65 for incident dementia (17), illustrating useful but limited screening discrimination and the possibility of misclassification. Because both ISI and the screening outcome are self-reported, common-method bias is also possible.

Third, several ISI domains have bounded ranges, ceiling effects, and restricted variability. Although HC3 robust standard errors confirmed the main temporal stability inference, HC3 does not remove limitations of the underlying score distribution, and future studies may consider models specifically suited to bounded or ordinal outcomes.

Fourth, residual confounding remains possible. Important unmeasured or incompletely measured factors include educational attainment, depressive symptoms, hearing or visual impairment, medication use, functional limitations, nutritional status, socioeconomic disadvantage, cognitive reserve, and vascular risk (38). Although chronic disease status was included as a baseline covariate, it was operationalized as a binary indicator of having at least one of the listed conditions and therefore could not capture the type, severity, duration, or cumulative burden of individual chronic or vascular conditions. In addition, the ISI independence domain may partly reflect broader functional and health-related characteristics rather than purely social engagement. Baseline assessment occurred in 2020, and COVID-19 restrictions may have influenced both social interaction and participant selection.

Fifth, approximately 39.2% of the 688-person baseline cohort was lost to follow-up. Retained-versus-lost comparisons indicated selective attrition. MI40 stabilized and truncated IPAW substantially improved balance on the observed baseline predictors and did not materially change the total-score inference, but inverse-probability weighting cannot correct selection driven by unmeasured variables or misspecification of the retention model.

Sixth, MICE relies on a missing-at-random assumption that cannot be empirically verified. The significant Little’s MCAR test indicates that MCAR is implausible but does not prove MAR. Increasing the final analysis to 40 imputations reduced Monte Carlo variability but cannot remove bias if missingness depends on unobserved values after conditioning on the imputation-model variables. The complete-case sensitivity analysis broadly supported the null total-score conclusion but differed for social curiosity, underscoring that exploratory domain estimates can be sensitive to missing-data handling.

Finally, generalizability may be limited because participants were community-dwelling older adults from one Japanese municipality. In addition, formation of the 2020 linked baseline cohort required participation in both separate same-period questionnaire forms (the ISI form and the cognitive-risk screening form). 157 individuals who did not complete one of these forms were not included in the linked baseline cohort, so selection could have occurred before the 688-person baseline cohort was formed.

## 6 Implications for policy and practice

The findings support cautious interpretation rather than a specific intervention prescription. After baseline cognitive screening status was accounted for, neither total social interaction nor any individual ISI domain showed a statistically significant independent association with later screen-positive cognitive impairment risk. Community social-engagement programs may have broad psychosocial value, but the present observational data does not establish that prioritizing a particular ISI domain will prevent cognitive impairment.

For policy and practice, the results primarily emphasize the importance of recognizing baseline cognitive vulnerability and of evaluating social-interaction interventions with designs capable of stronger causal inference. Future studies should incorporate more waves, objective cognitive measures, detailed socioeconomic and health confounders, and explicit evaluation of whether changes in social engagement precede within-person cognitive change.

## 7 Conclusion

Among community-dwelling older adults, baseline social interaction was not independently associated with subsequent screen-positive cognitive impairment risk after adjustment for baseline cognitive screening status in the final MI40 analyses. Secondary inverse associations for total ISI and social curiosity were attenuated in the principal models, and HC3, complete-case, standardized per-SD, and IPAW sensitivity analyses did not overturn the null total-score conclusion. These findings emphasize the importance of accounting for pre-existing cognitive differences and possible reverse directionality when interpreting longitudinal social–cognitive associations. More repeated measurements and objective cognitive assessments are required before causal or preventive implications can be drawn.

## Data Availability

The datasets presented in this article are not readily available because participant-level data cannot be publicly deposited due to participant privacy, municipal data-governance agreements, and consent restrictions. De-identified data may be made available to qualified researchers upon reasonable request, subject to review and approval by the data-holding institution. Data access is conditional on applicable ethical approval, execution of an appropriate data-use agreement, and authorization by the data-holding institution. Requests to access the datasets should be directed to Tokie Anme at tokieanme@gmail.com.
