# Supplementary material for: Longitudinal association between social interaction and screen-positive cognitive impairment risk in older adults: domain-specific effects and the impact of baseline cognitive status
Source: Front Public Health. 2026 Oct 6;14:1873956. doi: 10.3389/fpubh.2026.1873956 (PMC13680159; doi:10.3389/fpubh.2026.1873956)
Supplement: Supplementary file 1 [file Table_1.pdf]

The following supplementary analyses report the final 40-imputation (MI40) results used in the revised manuscript.

Table S1. Baseline comparison of retained participants and participants lost to follow-up

Variable	Retained (n = 418)	Lost (n = 270)	*p*	|SMD|
Age, years	73.13 ± 6.47	75.32 ± 7.75	< .001	0.313
ISI total score	16.48 ± 2.05	15.79 ± 2.77	.006	0.299
Independence	3.92 ± 0.43	3.84 ± 0.63	.061	0.167
Social curiosity	4.28 ± 1.06	3.98 ± 1.30	.004	0.260
Interaction	2.84 ± 0.45	2.70 ± 0.66	.004	0.259
Participation in the society	3.46 ± 0.72	3.30 ± 0.84	.025	0.216
Feeling of safety	1.86 ± 0.45	1.82 ± 0.50	.247	0.097
Sex, coded 1	45.9%	48.9%	.448	0.059
Living status, coded 1	90.6%	94.3%	.082	0.141
Subjective economic status, coded 1	81.5%	80.7%	.808	0.019
Care status, coded 1	8.2%	19.8%	< .001	0.335
Chronic disease status, coded 1	81.8%	84.8%	.315	0.081
Physical activity, coded 1	63.9%	54.0%	.014	0.202
Alcohol consumption, coded 1	36.7%	30.5%	.094	0.132
Smoking status, coded 1	8.4%	8.9%	.833	0.017
Baseline screen-positive cognitive risk	59.8%	62.3%	.529	0.050

Note. Continuous variables are shown as M ± SD and binary variables as percentages. SMD = standardized mean difference; absolute SMDs are reported.

Table S2. Original and inverse-probability-of-attrition-weighted cognitive-outcome sensitivity analyses

Exposure	Analysis	*OR* (95% CI)	*p*
Total ISI	Original	0.922 (0.787–1.079)	.309
Total ISI	Stabilized IPAW	0.896 (0.797–1.008)	.067
Total ISI	IPAW trimmed 1%–99%	0.901 (0.800–1.015)	.086
Independence	Original	0.596 (0.214–1.662)	.323
Independence	Stabilized IPAW	0.452 (0.137–1.486)	.191
Independence	IPAW trimmed 1%–99%	0.474 (0.145–1.552)	.218
Social curiosity	Original	0.814 (0.613–1.081)	.155
Social curiosity	Stabilized IPAW	0.777 (0.601–1.005)	.054
Social curiosity	IPAW trimmed 1%–99%	0.783 (0.605–1.014)	.064
Interaction	Original	1.134 (0.630–2.040)	.675
Interaction	Stabilized IPAW	1.039 (0.657–1.643)	.869
Interaction	IPAW trimmed 1%–99%	1.028 (0.650–1.627)	.905
Participation in the society	Original	1.029 (0.733–1.443)	.871
Participation in the society	Stabilized IPAW	0.983 (0.707–1.369)	.921
Participation in the society	IPAW trimmed 1%–99%	1.001 (0.714–1.402)	.997
Feeling of safety	Original	0.804 (0.456–1.416)	.449
Feeling of safety	Stabilized IPAW	0.874 (0.544–1.403)	.576
Feeling of safety	IPAW trimmed 1%–99%	0.863 (0.537–1.387)	.543

Note. IPAW = inverse probability of attrition weighting. Weighted models used stabilized weights; truncated analyses limited stabilized weights to the 1st–99th percentiles. All models adjusted for baseline screen-positive cognitive risk and baseline covariates.

Table S3. MI40 pooled HC3 analyses for follow-up social-interaction outcomes

Outcome	Baseline same-score B	95% CI	*p*	Baseline cognitive-status B	95% CI	*p*
ISI total score	0.620	0.498–0.742	< .001	−0.116	−0.388–0.157	.405
Independence	0.477	0.201–0.753	< .001	−0.037	−0.087–0.014	.157
Social curiosity	0.569	0.453–0.685	< .001	−0.180	−0.33 to −0.03	.018
Interaction	0.270	0.109–0.432	.001	−0.030	−0.115–0.056	.495
Participation in the society	0.394	0.281–0.507	< .001	0.001	−0.136–0.138	.991
Feeling of safety	0.394	0.190–0.598	< .001	−0.002	−0.072–0.068	.958

Note. B denotes an unstandardized regression coefficient. HC3 heteroscedasticity-consistent covariance matrices were estimated within each of 40 imputed datasets and pooled using Rubin's rules. All models also adjusted for the baseline covariates.

Table S4. Complete-case baseline-cognition-adjusted cognitive-outcome sensitivity analyses

Exposure	*OR* (95% CI)	*p*	N
Total ISI	0.860 (0.692–1.069)	.173	251
Independence	0.643 (0.239–1.726)	.380	313
Social curiosity	0.685 (0.478–0.981)	.039	304
Interaction	0.873 (0.438–1.742)	.701	304
Participation in the society	0.802 (0.501–1.283)	.357	266
Feeling of safety	0.759 (0.400–1.439)	.398	313

Note. Complete-case models used only participants with observed values for the outcome, exposure, baseline cognitive-risk indicator, and all baseline covariates.

Table S5A. Full pooled principal total-ISI cognitive-outcome model

Predictor	*OR* (95% CI)	*p*
Total ISI	0.922 (0.787–1.079)	.309
Baseline screen-positive cognitive impairment risk	8.051 (4.908–13.209)	< .001
Age	1.011 (0.969–1.054)	.628
Sex	0.619 (0.356–1.074)	.088
Living status	0.998 (0.428–2.327)	.996
Subjective economic status	0.649 (0.319–1.323)	.234
Care status	0.930 (0.355–2.437)	.882
Chronic disease status	1.108 (0.587–2.090)	.751
Physical activity	0.905 (0.527–1.552)	.716
Alcohol consumption	1.054 (0.601–1.850)	.854
Smoking status	0.564 (0.226–1.406)	.219

Note. Tables S5A–S5F. Estimates were pooled across 40 imputed datasets using Rubin's rules. Each model included the specified social-interaction exposure, baseline screen-positive cognitive impairment risk, and all baseline covariates. Binary covariates are interpreted for category 1 relative to category 0; operational definitions are provided in Supplementary Table S7. Odds ratios and 95% confidence intervals are reported to three decimal places; p values are reported to three decimal places, with p < .001 where applicable.

Table S5B. Complete pooled coefficients for the principal Independence cognitive-outcome model

Predictor	*OR* (95% CI)	*p*
Independence	0.596 (0.214–1.662)	.323
Baseline screen-positive cognitive impairment risk	8.175 (5.002–13.359)	< .001
Age	1.012 (0.970–1.055)	.591
Sex	0.613 (0.353–1.064)	.082
Living status	1.012 (0.433–2.365)	.978
Subjective economic status	0.607 (0.299–1.233)	.167
Care status	0.932 (0.356–2.440)	.886
Chronic disease status	1.106 (0.587–2.084)	.755
Physical activity	0.886 (0.520–1.509)	.656
Alcohol consumption	1.083 (0.617–1.899)	.782
Smoking status	0.590 (0.240–1.454)	.252

Table S5C. Complete pooled coefficients for the principal Social curiosity cognitive-outcome model

Predictor	*OR* (95% CI)	*p*
Social curiosity	0.814 (0.613–1.081)	.155
Baseline screen-positive cognitive impairment risk	8.028 (4.903–13.144)	< .001
Age	1.005 (0.963–1.050)	.814
Sex	0.629 (0.362–1.094)	.100
Living status	0.985 (0.421–2.308)	.973
Subjective economic status	0.639 (0.313–1.301)	.217
Care status	0.933 (0.356–2.446)	.888
Chronic disease status	1.096 (0.582–2.064)	.777
Physical activity	0.912 (0.533–1.561)	.736
Alcohol consumption	1.091 (0.621–1.916)	.763
Smoking status	0.564 (0.228–1.398)	.216

Table S5D. Complete pooled coefficients for the principal Interaction cognitive-outcome model

Predictor	*OR* (95% CI)	*p*
Interaction	1.134 (0.630–2.040)	.675
Baseline screen-positive cognitive impairment risk	8.622 (5.214–14.257)	< .001
Age	1.012 (0.970–1.055)	.588
Sex	0.621 (0.358–1.075)	.089
Living status	0.941 (0.408–2.172)	.887
Subjective economic status	0.602 (0.297–1.224)	.161
Care status	1.020 (0.397–2.625)	.966
Chronic disease status	1.098 (0.583–2.067)	.773
Physical activity	0.852 (0.500–1.452)	.556
Alcohol consumption	1.060 (0.603–1.861)	.840
Smoking status	0.618 (0.253–1.510)	.291

Table S5E. Complete pooled coefficients for the principal Participation in society cognitive-outcome model

Predictor	*OR* (95% CI)	*p*
Participation in society	1.029 (0.733–1.443)	.871
Baseline screen-positive cognitive impairment risk	8.423 (5.163–13.740)	< .001
Age	1.012 (0.970–1.056)	.584
Sex	0.615 (0.355–1.067)	.084
Living status	0.953 (0.414–2.195)	.910
Subjective economic status	0.612 (0.301–1.245)	.175
Care status	1.024 (0.394–2.659)	.962
Chronic disease status	1.094 (0.580–2.067)	.781
Physical activity	0.853 (0.501–1.454)	.560
Alcohol consumption	1.077 (0.609–1.905)	.799
Smoking status	0.620 (0.253–1.519)	.296

Table S5F. Complete pooled coefficients for the principal Feeling of safety cognitive-outcome model

Predictor	*OR* (95% CI)	*p*
Feeling of safety	0.804 (0.456–1.416)	.449
Baseline screen-positive cognitive impairment risk	8.317 (5.095–13.576)	< .001
Age	1.012 (0.971–1.056)	.564
Sex	0.607 (0.349–1.055)	.077
Living status	0.977 (0.422–2.263)	.957
Subjective economic status	0.634 (0.310–1.293)	.210
Care status	0.994 (0.387–2.552)	.990
Chronic disease status	1.086 (0.575–2.050)	.799
Physical activity	0.862 (0.508–1.464)	.582
Alcohol consumption	1.056 (0.601–1.855)	.850
Smoking status	0.593 (0.241–1.459)	.255

Table S6. Standardized per-SD odds ratios from principal baseline-adjusted models

Exposure	*OR* per 1 *SD*	95% CI	*p*
ISI total score	0.856	0.634–1.156	.309
Independence	0.806	0.525–1.236	.323
Social curiosity	0.805	0.597–1.085	.155
Interaction	1.058	0.813–1.375	.675
Participation in the society	1.022	0.790–1.321	.870
Feeling of safety	0.907	0.705–1.168	.449

Note. Each social-interaction exposure was standardized within each imputed dataset before fitting the baseline-cognition-adjusted logistic model. Estimates were pooled across 40 imputations.

Table S7. Original measurement categories and coding of baseline covariates used in the regression models

Variable	Coding	Analysis category definition	Original response categories / derivation rule
Sex	0/1	0 = female; 1 = male	Female and male categories were recorded in the baseline questionnaire; female was coded 0 and male 1.
Living status	0/1	0 = living alone; 1 = living with others	Question: household composition. Responses: 1 = living alone; 2 = couple only (spouse aged ≥65 years); 3 = couple only (spouse aged ≤64 years); 4 = two-generation household with son/daughter; 5 = other. Response 1 → 0; responses 2–5 → 1.
Subjective economic status	0/1	0 = economically difficult; 1 = ordinary/comfortable	Question: perceived current financial situation. Responses: 1 = very difficult; 2 = somewhat difficult; 3 = ordinary; 4 = somewhat comfortable; 5 = very comfortable. Responses 1‑2 → 0; responses 3‑5 → 1.
Care status	0/1	0 = no care/assistance need; 1 = care/assistance need	Question: need for care or assistance in daily life. Responses: 1 = no care/assistance needed; 2 = some care/assistance needed but not currently received; 3 = currently receiving some care/assistance (including care from family without formal long-term-care certification). Response 1 → 0; responses 2‑3 → 1.
Chronic disease status	0/1	0 = none; 1 = ≥1 listed condition	Question: any disease currently under treatment or with sequelae (multiple responses allowed). Options: 1 none; 2 hypertension; 3 stroke (e.g., cerebral hemorrhage/infarction); 4 heart disease; 5 diabetes; 6 hyperlipidemia/dyslipidemia; 7 respiratory disease (e.g., pneumonia/bronchitis); 8 gastrointestinal/liver/gallbladder disease; 9 kidney/prostate disease; 10 musculoskeletal disease (e.g., osteoporosis/arthropathy); 11 injury (e.g., fall/fracture); 12 cancer/malignancy; 13 blood/immune disease; 14 depression; 15 dementia (e.g., Alzheimer disease); 16 Parkinson disease; 17 eye disease; 18 ear disease; 19 other. Option 1 only → 0; any option 2‑19 → 1.
Physical activity	0/1	0 = inactive; 1 = active	Question: "Do you exercise?" Responses: 1 = almost every day; 2 = 3‑4 times/week; 3 = 1‑2 times/week; 4 = hardly ever. Response 4 → 0; responses 1‑3 → 1.
Alcohol consumption	0/1	0 = hardly ever/never; 1 = almost daily/sometimes	Question: "Do you drink alcohol?" Responses: 1 = almost every day; 2 = sometimes; 3 = hardly ever; 4 = never. Responses 1‑2 → 1; responses 3‑4 → 0.
Smoking status	0/1	0 = former/never; 1 = current smoking	Question: "Do you smoke?" Responses: 1 = smokes almost every day; 2 = smokes sometimes; 3 = used to smoke but quit; 4 = never smoked. Responses 1‑2 → 1; responses 3‑4 → 0.

Note. The category coded 0 served as the reference category for all binary covariates. Original Japanese questionnaire response categories are translated into English for reporting. For alcohol consumption, responses 1–2 ('almost every day'/'sometimes') were coded 1 and responses 3–4 ('hardly ever'/'never') were coded 0. For smoking, responses 1–2 (current smoking) were coded 1 and responses 3–4 (former/never smoking) were coded 0. Missing responses were handled according to the missing-data procedure described in the Methods.
